# The Prevention of Tuberculosis in Cattle

**Published:** 1909-05-22

**Authors:** 


### Public Health and Hygiene.

### THE PREVENTION OF TUBERCULOSIS IN CATTLE.

The relations of bovine to human tuberculosis, whatever view may be held as to the degree in which they operate in the spread of the disease, must now be taken as definitely established. It is even contended by competent observers that bovine tuberculosis as a factor in the diffusion of tubercular infection in man is of importance secondary to no other, and that in the efforts which are being made to limit the disease the control of tuberculosis in cattle is a measure of primary consideration. From whatever point of view the question is approached, whether from the merely economical or from the wider and more concrete hygienic standpoint, it must be admitted that the prevention of cattle tuberculosis is an achievement worthy of national endeavour. An attack, organised and enforced with all the resources of State effort is essential to success.

It is characteristic of English methods that the great municipalities should make the first essays in administrative action, and in the exercise of the powers which Parliament has granted them are to be found the chief attempts which have been made to secure food liable to infection free from tubercular taint In the absence of public abattoirs, and consequently of any efficient system of meat inspection, it has been found impossible to prevent the sale of tubercular meat, and although under the limitations to which they were subject the officers of the public health service have done much to lessen its consumption, it has to be admitted that the facilities for disposing of tubercular meat have been such as to leave no strong incentive for the farmer to eradicate the disease from his herd.

What are known as the model milk clauses for the prevention of the sale of tubercle infected milk have been incorporated in the private Acts of several of the more active municipalities, but these clauses are cumbersome to work, and cannot be said in effect to have been a serious check upon the sale of milk dangerously impregnated with tubercle bacilli. Doubtless the pressure which these clauses has exercised upon the minds of intelligent dairy farmers has done something to raise the problem of stamping out tuberculosis in cattle to one on the fringe of practical affairs, but so far as the great mass of dairy farmers in this country is concerned, the prevalence of bovine tubercle is an act of God with which only medical and veterinary faddists would have the temerity to interfere.

The Corporation of Birmingham, with commendable enterprise, has opened another chapter in the methods of tackling this difficult problem. About a year ago it appointed a committee to visit and report upon the dairy farms in Denmark, where methods had been adopted for stamping out tuberculosis in the herds. The committee was accompanied by Dr. John Robertson, the medical officer of health of Birmingham; and in the report of the committee and in a paper recently contributed to the Society of Medical Officers of Health are given the results of the inquiry. The committee in its visits to the Danish dairy farms had the advantage of the presence of Professor Bang, Principal of the Veterinary College at Copenhagen, and of Mr. Markeberg, Government Veterinary Inspector.

"The guiding principle governing Bang's method, as described by himself during the course of our visit, and observed by us in practical operation on the farms visited, is the gradual eradication of infection. He relies upon segregation and isolation, and not upon slaughter. Only cows with tuberculosis of the udder, and wasters manifestly dangerous to others from extensive open generalised tuberculosis, are slaughtered. Essentially his method depends upon (1) the use of tuberculin to diagnose the disease; (2) the complete separation of healthy animals from diseased; and (3) the gradual rearing up of a healthy non-infected stock to replace in due course the infected." By this method between 700 and 800 herds of cattle in Denmark have been freed from tuberculosis.

About a century ago Danish cattle are stated to have been free from tuberculosis, and the disease has been traced to the introduction of infected cattle from Switzerland, Schleswig, and Great Britain. The separation of the healthy from the diseased cattle out of doors is comparatively easily accomplished in Denmark, where it is customary to tether all cattle at grass. The separation indoors has been accomplished by the provision of a partition in the sheds free from a doorway or other opening and the restriction of each group to a separate end of the shed. The calves of tubercle-infected cows are immediately removed from their mothers and fed upon milk heated to 80° C. Herds freed from tubercle are tested twice a year with tuberculin and reactors are at once removed.

As a result of their visits of inspection and of the information they obtained in Denmark, the committee have arrived at the following conclusions:—

1. Bang's method has proved itself in Denmark during the past sixteen or seventeen years to be a practical and economical method of ridding herds of dairy cattle of tuberculosis.
2. They consider it to be a method which may equally well be introduced into this country with a probability of even better results.
3. Although slow in operation, the method has the advantage of not being costly and of causing little or no disturbance to the trade of the milk producer.

The Committee are further of opinion that all cases of open tuberculosis in cattle should be scheduled under the Contagious Diseases (Animals) Acts, and that veterinary assistance and a gratuitous supply of tuberculin should be placed at the disposal of all farmers undertaking to isolate reacting from healthy animals and prepared to keep their stock under satisfactory sanitary conditions.

If concurrently with these measures the larger municipalities will more vigorously exercise the powers with which they have been vested for preventing the sale of tuberculous food, we should see the dawn of a new era in English agriculture.

We have in Bang's method of stamping out tuberculosis in cattle a means of attaining a most desirable end which involves not so much an expenditure of money as the application of intelligence, a knowledge and appreciation of simple hygienic facts and a persevering industry which is assured of reward. No exception can be taken to exacting in full measure these essential qualities of success in an industry where their neglect threatens the public health. But it is certain that the inertia of the farmer will only be overcome if, while he is instructed and encouraged to abolish tuberculosis from his herds, he is at the same time penalised for the neglect of precautions necessary to obtain the end in view.

The condemnation of tubercular products, whether in meat or milk, were it universally insisted upon, would make it not worth while to permit the unrestrained perpetuation of the disease in animals, while it would undoubtedly reflect itself in improved health in man.

